# Trends in Comprehensive Abortion Care (CAC) and characteristics of women receiving abortion care in a tertiary hospital in Nepal

**DOI:** 10.1186/s12905-019-0739-9

**Published:** 2019-02-28

**Authors:** Jamuna Tamrakar Sayami

**Affiliations:** Cardio Thoracic Vascular and Transplant Center (MCTVC), Tribhuvan University Teaching Hospital Complex, Institute of Medicine, Tribhuvan University, Maharajgunj, Kathmandu, Nepal

**Keywords:** Comprehensive abortion care, Manual vacuum aspiration, Medical abortion, Post abortion care

## Abstract

**Background:**

One of the leading cause of maternal mortality and morbidity is unsafe abortion. Globally 55.7million of abortions occurred each year between 2010 and 2014. In lower resource countries 24.3 million abortions were unsafe which is significantly higher. Nepal is one of the lower resource countries among others. Comprehensive abortion care (CAC) service can reduce this burden among women.

**Methods:**

A retrospective review of CAC service register at Tribhuvan University Teaching Hospital (TUTH) was conducted to collect data from 2006 to2015 with approval from the Nursing Department to identify the trends of CAC service delivery, client characteristics, category of service providers, and reason for seeking CAC services, its effectiveness and complications. The data was entered in SPSS software and descriptive analysis was performed.

**Results:**

A total of 2367 women received CAC in ten years period showing similar trend as 272–275 cases per year. Women’s mean age was 28.4 years, 34% attained secondary level education and 98.9% were married. 70% were house wives and 84% multi gravid. The gestational period varied from 5 to 12 weeks. 85.6% had Manual Vacuum Aspiration (MVA) and 14.4% had Medical Abortion (MA). Only 37.6% women used any method of post abortion contraception. Unwanted pregnancy was the commonest reason for CAC. A majority of service providers were doctors (62.4%). The nurses were equally competent to provide CAC service as doctors.

**Conclusions:**

The number of women receiving CAC was relatively constant over the ten-year period. Nurses should be promoted for providing CAC services to cover a larger population in need.

## Background

Globally 55.7 million abortions occurred each year between 2010 and 2014. In low-resource countries, 78.2% of abortions are unsafe, compared to 17.8% in high-resource countries [1]. There was a sharp decline in maternal mortality, which fell from 580 maternal death per 100,000 live births in 1995 to 190 per 100,000 in 2013 with the abortion legalization [2]. In 2014 about 137,000 legal abortions were provided in Nepal. Public facilities provided 37, 34% by NGO facilities and 29% by private facilities. In 2014 an estimated 80,500 women were treated in health facilities for complications of miscarriage or induced abortion. Among the facilities providing post abortion care, the largest proportion was covered by the private sector [3]. In Nepal nearly 80% of maternal mortality was due to direct causes and out of which 13% were due to unsafe abortion [4]. A retrospective medical chart review of all gynecological cases was done at four large public hospitals in Nepal from 2001 to 2010; all cases of spontaneous and induced abortion complications were identified. Among all abortion cases, 23,493 cases of abortion complications identified [5]. The amendment of Muluki Ain (Civil Service Act) in September 2002 declared abortion as legal. Within this law, the woman can decide abortion on her own if she wished up to twelve weeks of conception, and up to eighteen weeks following a rape or incest. When the continuation of pregnancy possesses a serious threat to mother and if there is a major fetal congenital malformation incompatible with life, abortion can be done [6].

A range of providers, including nurses and midwives, have been shown to be competent to deliver abortion services safely in a number of settings. As with many other medical procedures, adherence to best practice standards ensures that the most effective and safest services are delivered in a respectful and sensitive manner that recognizes women as decision maker [7]. A randomized control trial in South Africa and Vietnam showed that the training of the mid-level service providers such as nurses to provide safe abortion services is the key for improving access. Study have shown that mid-level providers can provide Post Abortion Care (PAC) equally safely and effectively as physicians [8, 9]. To reduce the country’s high maternal mortality rate, access to safe abortion services has a high significance. Creating an enabling environment for women in the community and health facility to access the CAC services is crucial to reduce unsafe abortion in Nepal. The national safe abortion policy has been progressively implemented since CAC service was started first at Maternity Hospital, Kathmandu, Nepal in March 2004. According to the procedural order, both service providers and facilities must be approved and the policy sets out provision that both provider and facility must be registered with certificate of competency by concerned authority [10]. The elements of CAC are high quality abortion care service that women have access to and affordable in the communities where they live and work which includes contraception, post abortion care and pain management [11–13]. The World Health Organization (WHO) has recommended medical abortion, manual vacuum aspiration and dilatation and evacuation as safe methods of abortion. Similarly, the WHO clinical practice handbook for safe abortion also emphasizes about the provider’s awareness of local laws and reporting requirements within the national framework including all norms, standards and clinical practice related to abortion care. The provider should also possess the stated clinical skills to confirm diagnosis of pregnancy and preconditions of women to pursue termination of pregnancy and intervention method with the informed and voluntary decision making by the woman; nondiscrimination, confidentiality and privacy must be maintained [14]. The Who guideline on health role in providing safe abortion care and post abortion contraceptives states that a wide range of health care providers beyond the specialist doctors is an increasingly important public health strategy. The task shifting and sharing ensure a rational optimization of available health workforce to address shortages of specialized health care professionals, improve equity in access to health care and increase the acceptability of health services for those receiving them by utilizing Nurses, Auxiliary Nurse Midwives (ANMs) and complementary health care providers with necessary training [15]. Another study among 100 patients in Kathmandu Medical College Hospital (KMCH), Nepal, who received CAC services found that the major reason for abortion was no desire for more children (60%) followed by younger child or short interval in pregnancy (21%) [16].

## Methods

### Study setting

Tribhuvan University Teaching Hospital (TUTH) is a tertiary level referral teaching hospital situated in Maharajgunj, in the middle of Kathmandu, Nepal, as a part of Institute of Medicine, Tribhuvan University. There are twenty-eight departments in the hospital including Nursing, Dental, and Allied health sciences along with medical fraternity. Among specialty department, the Obstetrics and Gynecology department has the highest patient throughput in the hospital. Since 2006 CAC service was introduced in TUTH, family planning clinic as a part of government policy to expand CAC services in all health sectors. Since then the CAC service was provided here through trained doctor and Nurses.

Therefore, this study was done with the objectives to identify the trend of CAC service delivery, client characteristics, category of service providers, and reasons for seeking CAC services, its effectiveness and complications. This study was done to bring useful information to advocate for more involvement of nurses as CAC provider and scale up efforts to train nurses to provide quality CAC services.

### CAC procedure performed in the family planning clinic in the hospital

Standard CAC service is provided to those women who missed their menstrual period and seeking CAC service. The standard CAC service consists of counseling of the woman, then record of history, conduct physical and per vaginal examination at the time of visit. Prior to CAC service prophylactic antibiotic and analgesia (Doxycycline 100 mg and Ibuprofen 400 mg) used to be given half an hour before the procedure. Anesthesia is also given with 1% xylocaine Para cervical block. Manual Vacuum Aspiration (MVA) of the uterus is performed as described in standard techniques. After 1–2 h of observation, the woman is discharged with her choice of contraception (or contraception supplied to her)^.11^.

### Data collection and analysis

A retrospective review of CAC register was carried in Family Planning and CAC center of TUTH, Kathmandu starting from the period of inception, March 2006 until the end of March 2015 with the approval from the nursing department. The register of CAC service in the Family Planning and CAC center was explored for data collection. As the data were collected from the records, only variables available in the register could be analyzed. This include, name, age, gravid, parity, area of living, week of gestation, method of CAC procedure, CAC service provider, outcome of CAC procedure, complications and remarks. The information were first entered in the excel program and then transferred to SPSS software version 20, which was used for the analysis. Qualitative data included the reasons for abortion, complications which were analyzed manually.

### Ethical approval

Ethical approval (Ref no-37(6-11E) [2] /073–074) was obtained from the Institutional Review Board of Institute of Medicine, Tribhuvan University to conduct the study and publication of the manuscript. Since it was a retrospective review of the available information in the patient registry, individual participant’s consent was not applicable. Permission letter was also obtained from the TUTH for the publication of manuscript.

## Results

In total, there were 2367 women who received CAC services in the TUTH in ten years period. The women’s age ranged from 14 years to 53 years and the mean age was 28.4 years (SD = 5.8). The majority of women (54%) were young adults of age 20–29 years. Regarding the level of education the highest proportion (34%) had secondary level education while only (4.6%) had masters level. Similarly, 70% of the women were house wives, 56.2% were non classified ethnic group (Brahmin, Chhetris, Thakuri, Sanyasi Marwadi etc., Health Management Information System (HMIS)-11) who are considered as educated rich and upper class in the social system and 88.8% were from the Kathmandu valley. The majority (98.9%) of the women were married (Table 1).

**Table 1:** Demographic characteristics of CAC service users

Variables	Number	Percent
Age (*n* = 2357)		
14-19 years	94	4
20-29 years	1284	54.2
30-39 years	876	37.2
40–49 + years	106	4.4
Education (*n* = 2315)		
Illiterate	358	14
Literate	546	25.4
Basic (ECD- grade8)	165	7
Secondary [9–12]	788	34
Bachelor	350	15
Master	108	4.6
Occupation (*n* = 2309)		
Housewife	1637	70.8
Agriculture	6	0.2
Service	397	17
Student	146	6.5
Own business	123	5.5
Ethnicity (*n* = 2163)		
Hill Dalit (1a)	17	0.8
Terai Dalit (1b)	9	0.4
Disadvantaged Hill Janajati (2a)	342	16
Disadvantaged TeraiJanajati (2b)	129	6
Disadvantaged non-dalitgroup [3]	44	2.0
Religious minority [4]	13	0.6
Relatively advantaged Janajati [5]	382	18
Non-classified ethnic groups [6] (Brahmin, Chhetris, Thakuri, Sanyasi Marwadi etc., HMIS-11)	1218	56.2
Residence of CACusers (*n* = 2266)		
Inside Kathmandu	2013	88.8
Outside Kathmandu	253	11.2
Maritalstatus (*n* = 2367)		
Married	2342	98.9
Unmarried	25	1.1

### Reproductive health status of CAC users

Reproductive health status of women showed multi gravid 84% while 16% were primi gravid. Similarly, parity of women showed 63% were multi para. The gestational week of conception varied from 5 to 12 weeks. Eighty six percent of women had MVA as method of abortion and 14% had medical abortion. Regarding the post abortion contraception, only 37.6% women used any method, among them, the 29%, used Intra Uterine Device (IUD) followed by Depo-Provera 28% (Table 2).

**Table 2:** Reproductive health status of CAC users

Variable description	Number	Percent (%)
Gravid (*n* = 2354)		
Primi	381	16
Multi	1973	84
Para (*n* = 1945)		
Primi	717	37
Multi	1228	63
Gestational Weeks (n = 2367)		
5- 8 weeks	2098	88.6
9-12 weeks	269	11.4
Procedure of CAC (n = 2367)		
Manual Vacuum Aspiration(MVA)	2027	85.6
Medical abortion [7]	340	14.4
Used post CAC contraception (*n* = 894)		
IUD	258	29
Depo	253	28
Oral contraceptive Pills	132	**15**
Norplant	22	2
Natural method	18	2
Condom	125	14
Plan for Non Scalpel Vasectomy	44	5

### Service provider and reasons for seeking CAC

**Fig. 1:**
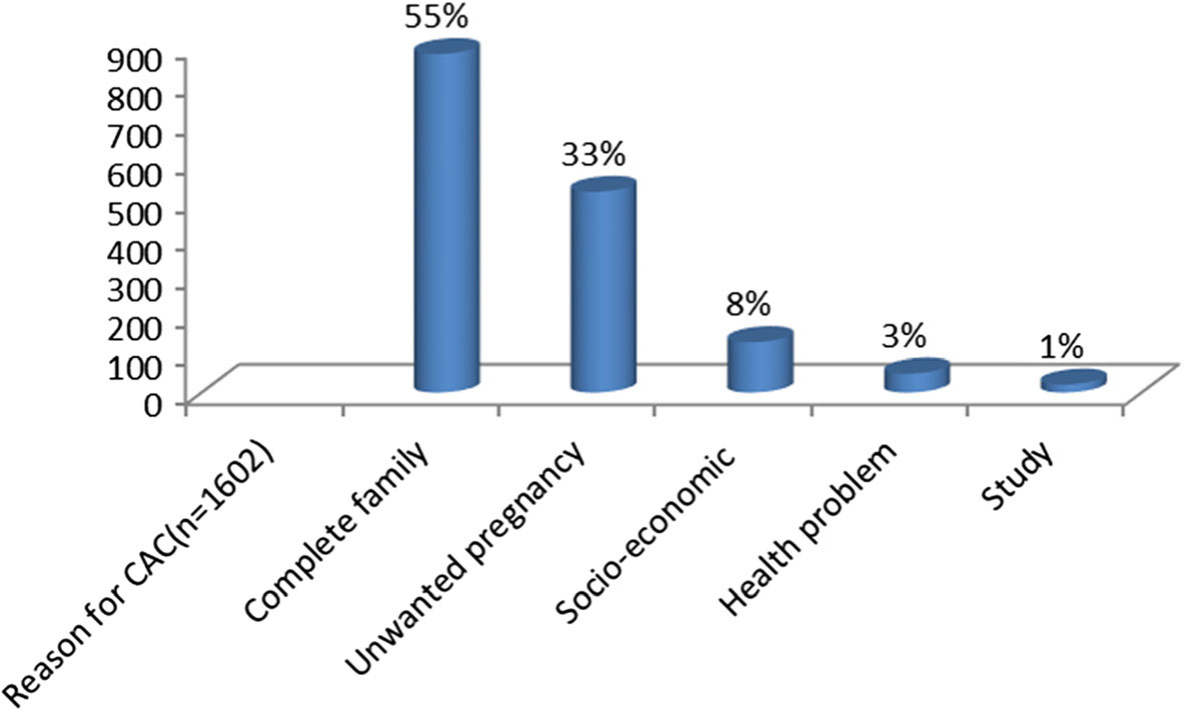
Reasons for using Comprehensive Abortion Care services

More than half of the CAC service providers in TUTH were doctors. The reason for using the CAC services were completed family (55%) among multi parous women, unwanted pregnancy (33%), socioeconomic (includes financial problem, family problem, small child, rape, and husband left) along with other problems (8%) (Fig. 1).

### Trends of CAC services in TUTH

**Fig. 2:**
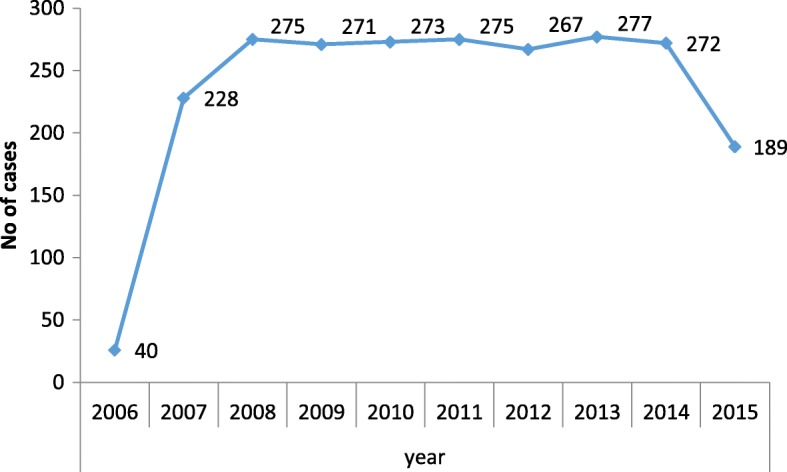
Trend of CAC in TUTH 2006-2015 AD

The trend of CAC service delivery in ten years from 2006 to 2015 showed mostly similar patterns except in the first year 2006, there was 40 cases only as it was the beginning of service year only few months was covered. In following years the CAC service pattern ranged from 275 to 272 cases per year. In 2015 the CAC service significantly reduced to 189 cases only compared to previous years (Fig. 2).

### Providers and procedures used in CAC service

**Fig. 3:**
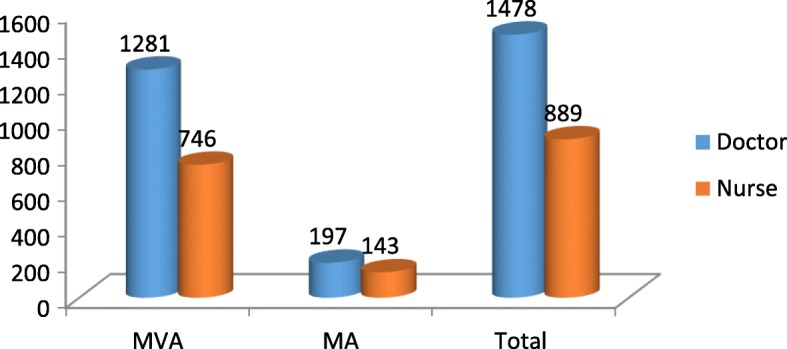
Providers and procedure used for CAC service

In the initial period of introduction of the CAC service, it was provided by doctors only and nurses were trained as assistants. Later, nurses were also trained as provider of CAC service for dealing with pregnancies up to eight weeks gestation period. Doctors provided 62% (*n* = 1478) CAC services out of which 1281 was Manual Vacuum Aspiration (MVA) and 197 was Medical abortion (MA). Nurses provided 38% (*n* = 889) CAC services and out of which 746 was MVA and 143 was MA (Fig. 3).

### Complications of CAC services at TUTH

**Fig. 4:**
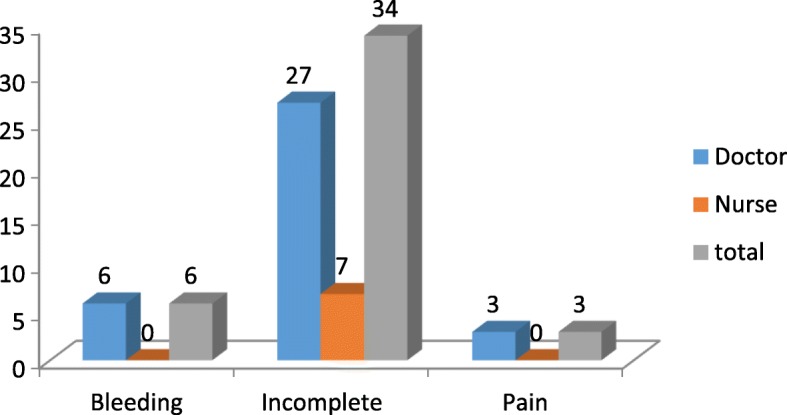
Complications of CAC Procedure

Overall incidence of complications of CAC services was 1.8%. The commonest complication was incomplete abortion representing 1.4% which was again intervened with Post Abortion Care procedure (PAC). Six women had bleeding complications which was treated with ringer’s lactate IV fluid and other measures such as rest and observation and misoprostol 4 tabs. Vaginal insertion in follow up. Three women complained abdominal pain and were managed with pain medication. One woman got continuation of pregnancy which was identified in the follow-up. Regarding the rate of complications among the service providers, it showed that the rate of CAC complications was 2.4% among doctors and 0.8% among nurse providers (Fig. 4).

## Discussions

The trend of CAC service delivery in ten years from 2006 to 2015 showed a similar pattern in different years. In 2015 the CAC service significantly reduced to 189 cases. This may be due to great earthquake in the year 2015 when client attendance and movement was limited in the CAC center or they were seeking CAC service from the other nearby CAC delivery sites to them.

Reproductive health status of women who used CAC service showed multi gravid 84% while 16% were primi gravid. And the parity of women showed 63% were multi para.

Eighty-six percent of women had MVA as method of abortion and 14% had medical abortion who seeks CAC services. This showed the high prevalence of unwanted pregnancy among the married women which needs to be addressed with appropriate family planning (FP) counseling. A retrospective study in CAC and PAC center of a maternity hospital in Nepal showed that the major reason for seeking abortion was too many children (59%) followed by illegitimate pregnancy (16%) [15]. However, this study showed the reason for seeking CAC was completed family (55%) followed by unwanted pregnancy (33%). Regarding the contraceptive devices used by women, previous study showed that contraceptive acceptance rate was 90% [16], however it was only 38% in this study. This finding is an indicator that much greater efforts are needed to increase the delivery of contraception to women immediately following the abortion procedure. Although CAC was safe in majority of women, some women were found to have complications. Thus an unwanted pregnancy must be avoided rather than being exposed to the risks of abortion.

A study done at southern general hospital, Glasgow, UK, found that 12% of abortion recipients’ were adolescents among them 18–19 years of age accounted for 8% and 15–17 years of age was 3% and those younger than 15 years of age was 0.2% in 2014 [17]. However, only 4% of women were adolescents in this study. Regarding the complications of CAC, this study showed that the overall prevalence was1.8%. The commonest complication was incomplete abortion (1.4%); six women had bleeding complications and three women complained abdominal pain which were comparatively lower than the reports of other studies. In other settings of low resource countries the complication rate of incomplete abortion was 1.3% which was higher than expected range of 0.5–1.2% [18]. Thus, it is suggested to develop provision of adequate training to providers for ensuring the complete removal of retained product of conception (RPOC) after the CAC procedure. Likewise, to aid in expulsion of small fragments of RPOC not removed during the procedure, administration of oxytocin, should be made as a rule [19]. Similarly, use of transvaginal ultrasound after the CAC procedure may help in determining whether products of conception remain in uterus or not [20, 21].

Similarly regarding the rate of complications among the service providers it showed that the rate of CAC complications was 2.4% among doctor providers and 0.8% among nurse providers. This showed that nurses are equally competent to the doctors in this study. This may be because nurses performed CAC only up to 8 weeks pregnancy while doctors do up to twelve weeks of pregnancy. This may be further influenced by the fact that the doctors were resident trainees in the most of the cases. In this regard a study result from Maternity hospital at Kathmandu showed that 1.6% (*n* = 68) of women who had surgical abortion and 1.2% (*n* = 12) of women who had medical abortion women had experienced complications, the overall complication rate being1.4% [22]. Clients preferred nurses to physicians by 67%. Nurses and facility managers pointed out a need for additional support, including further training and improve drug and equipment supply [19]. To maintain service quality for effective abortion care there should be a provision of continuous service improvement strategies to meet the need and rights of women [23]. Substandard service quality results in low acceptability of legal abortion services that may lead women to seek unqualified providers to perform self-induced abortions. This may result in abortion-related morbidity and mortality [20, 21]. The result of a multicenter randomized controlled equivalence trial done in five rural district hospitals in Nepal stated that the provision of medical abortion up to 9 weeks’ gestation by mid level providers (staff nurses (3-year degree) and auxiliary nurse midwives) and doctors was similar in safety and effectiveness. Appropriately trained midlevel health-care providers can provide safe, low-technology medical abortion services for women independently from doctors. A systematic review concluded that the medical termination of pregnancy (TOP) and medical treatment of incomplete miscarriage performed by trained non doctor providers in the first trimester is probably as safe as treatment provided by doctors. They also concluded that women were equally satisfied with their providers regardless of who treats or manages their medical TOP [24, 25].

The current study was done with limited data available in the CAC register so could not analyze variables which were done in reference studies.

## Conclusions

The trend of CAC service use in TUTH is nearly constant in ten years period except some decline in the year 2015. The major reason for undergoing CAC service was unwanted pregnancy. This is not a rigorous scientific comparison of complication rates for doctors and nurses and therefore could not draw the conclusion that nurses perform CAC as safely as doctors. This is an observational retrospective study that identified a lower complication rate for nurses than doctors. Additional training and supply of drug and equipment are needed to strengthen CAC service and ensure that nurses are able to continue providing high quality care. Training of nurses should be scaled up to expand CAC service provision across Nepal.

## Abbreviations

ANMs: Auxiliary Nurse Midwives
CAC: Comprehensive Abortion Care
FP: Family Planning
HMIS: Health Management Information System
IOM: Institute of Medicine
KMCH: Kathmandu Medical College Hospital
MA: Medical Abortion
MVA: Manual Vacuum Aspiration
PAC: Post Abortion Care
RPOC: Retained Product of Conception
SPSS: Statistical package for Social Science
TOP: Termination of Pregnancy
TUTH: Tribhuvan University Teaching Hospital
WHO: World Health Organization
